# Freeze-Drying Technology in Foods

**DOI:** 10.3390/foods9070920

**Published:** 2020-07-13

**Authors:** Valentina Prosapio, Estefania Lopez-Quiroga

**Affiliations:** School of Chemical and Engineering, University of Birmingham, Birmingham B15 2TT, UK; E.Lopez-Quiroga@bham.ac.uk

**Keywords:** freeze-drying, process design, rehydration, modelling, microstructure, food quality, encapsulation, processes combination

Freeze-drying (or lyophilisation) is a drying method, largely employed in the food industry. It consists in the freezing of the product, followed by sublimation of the ice at reduced pressure. Among drying processes, freeze-drying is considered to be the gentlest one, as it causes negligible damage to the product microstructure, allowing fast rehydration rates and high rehydration capacity, and good preservation of the physical chemical properties.

Despite being a common technique, lot of research on freeze-drying is still ongoing to optimise the process conditions according to specific applications, to improve the product characteristics by applying pre-treatments, and to reduce the energy costs and processing time. 

The Special Issue “Freeze-Drying Technology in Foods” focuses on the application of freeze-drying on food and nutraceutical fields and groups four original studies and one review. 

The study carried out by Silva-Espinoza et al. [1] focused on the optimisation of the freeze-drying operating conditions to better preserve the physical-chemical properties of orange puree. The authors investigated the effect of freezing rate (conventional and blast freezer), working pressure (5–100 Pa) and shelf temperature (30–50 °C) on quality parameters such as colour, porosity, mechanical properties, water content, vitamin C, total phenols, β-carotene and antioxidant activity. Colour analyses showed that colour was better preserved when the highest operating pressure, highest temperature, and fast freezing conditions were used. Freeze-dried purees showed a high degree of porosity, but sample porosities obtained at different conditions were not statistically different. Mechanical analysis showed that the higher mechanical resistance of the sample is achieved working at the lowest pressure and the highest temperature. In addition, samples with high mechanical resistance to fracture showed the smallest moisture content. Lower degradation of the nutrients was then observed at a higher temperature due to the faster completion of the drying process. The authors concluded that the optimal freeze-drying conditions to maximise the quality of freeze-dried orange puree are low pressure and high temperature.

Munzenmayer et al. [2] studied the effect of applying CO_2_ laser microperforations to blueberry skin prior freeze-drying. The results showed that the primary drying time was significantly reduced from 17h for non-treated berries to 13h when nine microperforations per berry were applied, with minimal effect on the fruit appearance. At the same time, the fruit quality was also significantly improved, as the percentage of non-busted blueberries at the end of the process increased from 47% to 86%. In fact, the authors showed that the microholes work as pathways for the escape of vapour from the sublimating front through the weakened mass transfer resistance of the blueberry skin, relieving the pressure development underneath, eventually avoiding the fruit bust and improving the quality of the resulting product with a reduced processing time. 

Prosapio et al. [3] applied freeze-drying to gellan gun gels loaded with vitamin B_2_ and investigated the effect of the gel pH on drying and release kinetics. They observed that acidified gellan gum gels at pH 2.5 showed the fastest drying rate, whereas gels at pH 4 showed the slowest rate. For natural pH samples (5.2), the Page model provided the most accurate description of freeze-drying kinetics, whereas the Wang and Singh model predicted more accurately the kinetics at pH 4 and 2.5. The authors investigated also the effect of the gel pH on the vitamin release mechanism using the Korsmeyer–Peppas model. Freeze-dried gels at pH 4 completed the vitamin release in about 9.5 h; gels at natural pH in 6h, while samples at pH 2.5 in 3 h. These differences were ascribed to the different gel microstructure. Freeze-dried gellan gum gels at pH 4 exhibited an aggregated and rigid structure that can impede mass transfer within the gel, increasing the time needed to release the vitamin completely from the substrate. Samples at pH 2.5 presented a low aggregated and weak structure that lead to breakage during the release experiments making the vitamin delivery faster. Natural pH gels showed an intermediate behaviour due to an intermediate level of aggregation. The Korsmeyer–Peppas model was used to analyse experimental release curves, revealing that samples at pH 5.2 display a typical Fickian behaviour, while acidified samples at pH 4 have combined both Fickian and relaxation mechanisms.

He et al. [4] investigated the emulsion properties of Aquafaba (AQ), a viscous by-product solution produced during cooking chickpea or other legumes in water. They carried out a screening on the different chickpea cultivars grown in Canada (CDC Leader, CDC Orion, CDC Luna, CDC Consul and Amit) and studied the impact of chickpea seed physicochemical properties (Seed coat incidence, Seed dimensions, surface area per unit mass of seed and seed coat weight per surface area) and hydration kinetics on the properties of AQ-based emulsions. The authors showed that the type of cultivar has a significant effect on the emulsion capacity and stability, being these values the highest when the CDC Leader cultivar was employed. In contrast, a correlation between the composition of the chickpea seed (carbohydrates, proteins, fat) and the emulsion properties was not observed. 

Bhatta et al. [5] conducted a review on the application of freeze-drying to plant-based foods which presents the most recent research publications on the subject and also includes original research on the topic by the authors. This work recalls the principle of freeze-drying and the main features of plant-based foods, highlighting their advantages and challenges upon lyophilization, and offers a thorough overview of the applications of freeze-dried fruits, vegetables and speciality foods (coffee, tea, spices). It follows a discussion of the most common studied materials and the effect of the process conditions (shelf temperature, pressure, processing time, sample size/cut) on product quality (shrinkage, porosity, colour, rehydration, nutrients retention, moisture content, thermal properties, morphology, texture, powder flowability). Finally, and with the aim of revealing potential improvements to product quality and process optimization in plant-based foods, the authors carried out a critical analysis of the most common pre-treatments: (i) chemical, i.e., immersion of the product in alkaline or acid solutions of oleate esters prior to drying; (ii) mechanical, consisting in the peeling, abrasion of the surface, puncturing the skin, or cutting the fruit in various shapes; (iii) thermal, involving blanching or steaming; (iv) freezing pre-treatments, including individual quick freezing, freeze-granulation, foaming, and the application of infrared energy, ultrasounds and microwaves. 

In summary, the five papers published in this Special Issue highlighted the main challenges in the ongoing research on freeze-drying and identified strategies to make this method more convenient and to better preserve the product quality with the aim of meeting industry needs and consumer expectations.
